# Tuning the Compatibilizer Content and Healing Temperature in Thermally Mendable Polyamide 6/Cyclic Olefin Copolymer Blends

**DOI:** 10.3390/polym17030280

**Published:** 2025-01-22

**Authors:** Davide Perin, Luigi Botta, Daniele Rigotti, Andrea Dorigato, Giulia Fredi, Alessandro Pegoretti

**Affiliations:** 1 Department of Industrial Engineering, University of Trento, Via Sommarive 9, 38123 Trento, Italy; daniele.rigotti-1@unitn.it (D.R.); andrea.dorigato@unitn.it (A.D.); alessandro.pegoretti@unitn.it (A.P.); 2 National Interuniversity Consortium of Materials Science and Technology (INSTM), Via Giuseppe Giusti, 9, 50121 Florence, Italy; luigi.botta@unipa.it; 3 Department of Engineering, University of Palermo, Viale Delle Scienze, 90128 Palermo, Italy

**Keywords:** polyamide 6, cyclic olefin copolymer, polymer blends, rheology, fracture toughness, self-healing

## Abstract

This study presents the formulation and comprehensive characterization of compatibilized polyamide 6 (PA6)/cyclic olefin copolymer (COC) blends with the aim of developing a self-healing matrix for thermoplastic structural composites. Rheological analysis highlighted the compatibilizing effect of ethylene glycidyl methacrylate (E-GMA), as evidenced by an increase in viscosity, melt strength (MS), and breaking stretching ratio (BSR), thus improving the processability during film extrusion. E-GMA also decreased COC domain size and improved the interfacial interaction with PA6, which was at the basis of a higher tensile strength and strain at break compared to neat PA6/COC blends. E-GMA also significantly boosted the healing efficiency (HE), measured via fracture toughness tests in quasi-static and impact conditions. The optimal healing temperature was identified as 160 °C, associated with an HE of 38% in quasi-static mode and 82% in impact mode for the PA6/COC blends with an E-GMA content of 5 wt% (PA6COC_5E-GMA). The higher healing efficiency under impact conditions was attributed to the planar fracture surface, which facilitated the flow of the healing agent in the crack zone, as proven by fractography analysis. This work demonstrates the potential of E-GMA in fine-tuning the thermomechanical properties of PA6/COC blends. PA6COC_5E-GMA emerged as the formulation with the best balance between processability and self-healing efficiency, paving the way for advanced multifunctional self-healing thermoplastic composites for structural applications.

## 1. Introduction

Thermoplastic structural composites have become interesting for many engineering applications because, in addition to their exceptional strength-to-weight ratio, corrosion resistance, and design flexibility typical of all polymer composites [1,2], they also exhibit higher toughness, ease of processing, and recyclability [3,4,5]. Nevertheless, a major drawback of all composites is their vulnerability to damage, which may result in reduced structural integrity and a shorter lifespan [6]. A promising strategy to overcome this issue is the development of self-healing thermoplastic composites.

Self-healing mechanisms integrated into composites can be divided into two broad categories: extrinsic and intrinsic. Extrinsic systems exploit the inclusion of healing agents such as microcapsules or hollow fibers [7,8]. Upon damage or critical solicitation, healing agents are released to repair the damaged materials. These agents typically contain reactive chemicals such as epoxy resins or monomers that polymerize and seal cracks when activated by damage [9]. On the other hand, intrinsic systems exploit the reversible nature of certain chemical bonds or supramolecular interactions within the polymer matrix [10]. Dynamic covalent bonds, such as Diels–Alder reactions [11,12] and disulfide bonds [13], can break and reform, enabling the material to heal itself [14]. Additionally, self-healing systems can be non-autonomous or autonomous, that is, requiring or not requiring an external trigger for the healing process to occur.

Although the self-healing process has been investigated mainly for thermosetting matrices, the production of thermoplastic self-healing composites can also be very interesting. The addition of self-healing functionality to high-performance thermoplastic matrices can further improve the damage tolerance, ease of repair, processing flexibility, and lifespan without compromising their recyclability potential. Thermoplastic polymers can be engineered to exhibit self-healing properties through mechanisms such as the shape memory effect [15], thermally reversible crosslinking [16], and the addition of thermoplastic healing agents [17]. The last mechanism has been thoroughly investigated for thermosetting matrices [18,19], while little to no literature has been found on the addition of thermoplastic healing agents to a thermoplastic matrix.

The fundamental aspect of self-healing thermoplastic composites lies in the careful selection of the thermoplastic matrix because it provides a structural framework and plays a critical role in the self-healing process [20,21]. The choice is guided by the mechanical properties, thermal behavior, and compatibility with other composite constituents. Advanced engineering techniques, including molecular design and blending, have been employed to enhance the intrinsic properties of the matrix, thereby enabling effective self-repair [22]. However, the incorporation of self-healing functionalities into thermoplastic matrices often involves integration of different phases or components. To ensure a homogeneous distribution and robust interfacial bonding between the dispersed healing agent and the matrix, the use of compatibilizers is crucial [23,24]. Compatibilizers are additives that enhance the miscibility and adhesion of immiscible components within the matrix [25,26]. They facilitate the dispersion of self-healing agents and improve the overall mechanical performance of the material. Common compatibilizers include block copolymers [27], graft copolymers [28], and reactive coupling agents [29], and they should be selected based on blend constituents.

Recently, our group has focused on the production of thermoplastic polyamide 6 (PA6)-based self-healing matrices containing various amounts of polycaprolactone (PCL) [30] or cyclic olefin copolymer (COC) [31] as healing agents. The most promising system was PA6/COC (70/30 wt%/wt%), which was then combined with different compatibilizers (i.e., poly(ethylene)-graft-maleic anhydride (PE-g-MAH), polyolefin elastomer-graft-maleic anhydride (POE-g-MAH), and ethylene glycidyl methacrylate (E-GMA)), added in a weight fraction of 5 wt% to improve the dispersion of the healing agent and, thus, the mechanical properties and healing efficiency of the resulting blends [32]. The most promising compatibilizer, i.e., the one that yielded a higher healing efficiency, was E-GMA. This was because E-GMA could form covalent bonds with PA6, because the epoxy groups of E-GMA can react with both the amine and the carboxyl end groups of PA6. In contrast, E-GMA likely interacts mostly through weak intermolecular interactions with COC, through the ethylene segments. Thus, the interfacial adhesion and dispersion were enhanced without hindering the healing flow of the COC in the crack zone. This is better explained in our previous work [32]. Although promising, these results are far from being optimized because only a single weight fraction of E-GMA (5 wt%) and a single healing temperature (140 °C) were investigated in that study.

Hence, this work represents a further advancement in the development of thermoplastic self-healing matrices for structural composites. The aim is to optimize a promising system, i.e., PA6/COC (70/30 wt%/wt%), by determining the optimal amount of E-GMA (1, 3, and 5 wt%) and the most suitable healing temperature (120, 140, 160, and 180 °C). An in-depth examination of the rheological properties was conducted using dynamic and capillary rheometry to understand the processability of the blends. Then, the selected systems were subjected to in-depth mechanical characterization and evaluation of the self-healing capability through quasi-static and impact fracture toughness tests. The differences in the healing capability between these testing modes were explained in light of an accurate microstructural analysis to correlate the healing efficiency with the fracture surface morphology.

## 2. Materials and Methods

### 2.1. Materials

The polyamide 6 was a Radilon S 24E 100 NAT, kindly provided by Radici Group SpA (Gandino, Italy) in pellet form (density = 1.14 g/cm^3^; melting temperature = 220 °C). The healing agent was a commercial-grade COC known as Topas COC 9506F-500 (TOPAS Advanced Polymers GmbH, Raunheim, Germany), delivered in pellet form (density = 1.01 g/cm^3^; glass transition temperature (T_g_) = 65 °C; norbornene content = 61 wt%). E-GMA compatibilizing agent was purchased from Merck KGaA (Darmstadt, Germany) in the form of granules (melt flow index (MFI) at 190 °C, 2.16 kg = 5 g/10 min; glycidyl methacrylate content = 6.5–9.0 wt%).

### 2.2. Sample Preparation

To avoid hydrolytic degradation during the melt mixing operations, PA6 granules were dried at 80 °C for 12 h in a vacuum oven, whereas the COC and E-GMA pellets were dried at 50 °C in a ventilated oven for 12 h. Initially, both PA6 and COC granules were melt-compounded with a constant PA6/COC weight ratio of 70/30, in a Thermo Haake Rheomix 600 internal mixer equipped with counter-rotating rotors operating at 60 rpm, at a temperature of 230 °C for 1 min. Subsequently, the addition of 1, 3, or 5 wt% compatibilizer was performed for a total processing time of 6 min. The PA6/COC ratio was selected according to a previous study by our group [31], and the compatibilizer concentration was varied to determine the optimal E-GMA concentration. The compatibilized blends were subsequently compression-molded in a Carver hot plate press at 235 °C for 8 min under an applied pressure of 3.4 MPa. Using this methodology, square sheets of different thicknesses (120 × 120 × 2 mm^3^ and 100 × 100 × 5 mm^3^) were prepared. The non-compatibilized blend is denoted as PA6COC, while the compatibilized blends are labeled as PA6COC_x_E-GMA, where x represents the concentration of the compatibilizer. Table 1 lists all the prepared samples and the corresponding weight fractions of each constituent.

### 2.3. Characterization

#### 2.3.1. Rheological Properties

Dynamic rheological measurements were conducted using an HR-2 Discovery Hybrid Rheometer (TA Instruments, New Castle, DE, USA) operating in a parallel-plate configuration (plate diameter = 25 mm, gap = 1 mm). Frequency sweep tests were performed at five different temperatures: 230, 240, 250, 260, and 270 °C, in air, in a frequency range of 0.05 to 600 rad/s, applying a strain amplitude of 1%. Through these tests, it was possible to determine the trends of storage modulus (G′), loss modulus (G″), and complex viscosity (η*) as a function of the frequency. A minimum of three specimens were tested for each composition. Capillary rheological measurements were performed using a Rheological 1000 instrument (CEAST, Pianezza, Italy). The capillary diameter was 1 mm, the length-to-diameter ratio was 40, and the testing temperature ranged from 220 to 230 °C. The rheological behavior in non-isothermal elongational flow was assessed using the same capillary rheometer equipped with a tensile drawing system. The extruded filament passed through a pulley system rotating at an initial speed and was then drawn by two counter-rotating rolls at constant acceleration. The test ended with the filament breaking. The force at break in the molten filament was considered the melt strength (MS). The breaking stretching ratio (BSR) was calculated as the ratio of the drawing speed to the extrusion speed at the filament breakage. Each rheological characterization was performed at least thrice for each composition and temperature.

#### 2.3.2. Microstructural and Chemical Properties

Field emission scanning electron microscopy (FESEM) micrographs of the cryofractured surface of the virgin and healed samples were acquired using a Zeiss Supra 40 microscope operating at an acceleration voltage of 2.5 kV. A platinum/palladium (80:20) conductive coating was sputtered onto the specimens before observation to render them electrically conductive.

#### 2.3.3. Mechanical Properties

Quasi-static tensile tests were performed at ambient temperature using an Instron^®^ 5969 tensile testing machine (Instron, Norwood, MA, USA) equipped with a 1 kN load cell, and ISO 527-1 1BA specimens with a gauge length of 25 mm. The tests were performed at a crosshead speed of 10 mm/min, and at least ten specimens were tested for each composition. Quasi-static tests were used to determine the maximum stress (σ_max_) and strain at break (ε_b_). To determine the elastic modulus (E), quasi-static tensile tests were performed using the same machine equipped with an Instron^®^ 2620-601 extensometer, with a gauge length of 12.5 mm, at a crosshead speed of 0.25 mm/min. As reported in the ISO 527-1 standard, the elastic modulus was calculated as the secant modulus considering the stress levels associated with strain values of 0.05% and 0.25%. A minimum of five specimens were tested for each formulation.

The fracture toughness of the compatibilized blends was evaluated using single-edge notched bending (SENB) specimens with dimensions of 44 × 10 × 5 mm^3^, a sharp notch 5 mm in depth, and a span length of 40 mm. Tests in the quasi-static mode were performed according to the ASTM D5045 standard, using an Instron^®^ 5969 electromechanical testing machine (Instron, Norwood, MA, USA). Three-point bending tests on notched specimens were performed at a crosshead speed of 10 mm/min, and at least 12 specimens for each composition were tested. Tests in impact mode were performed using an instrumented CEAST impact machine equipped with a hammer with a mass of 0.5 kg, imposing a starting angle of 60° and an impact speed of 1.5 m/s. A minimum of 12 specimens were tested for each formulation. From the load–displacement curves, the maximum load sustained by the samples ($P_{MAX}$) was determined, and it was thus possible to determine the critical stress intensity factor (K_IC_), both in quasi-static and impact conditions, according to the expression reported in Equation (1),

(1) $$K_{IC}=\frac{P_{MAX}}{BW^{1/2}}\;\cdot f(x)$$

where B is the thickness of the samples, W is their width, and $f\left(x\right)\;$ is a calibration factor defined by the ASTM D5045 standard, being x=a/W the ratio between the notch depth (a) and the width of the specimens. From the integration of the load–displacement curves and the evaluation of the system compliance, the critical strain energy release rate (G_IC_) values in quasi-static mode were calculated, according to the expression reported in Equation (2),

(2) $$G_{IC}=\frac{∆U}{BW\varphi }$$

where ∆U is the difference between the total energy absorbed by the specimens and the energy absorbed in the indentation tests, while φ is an energy calibration factor, whose expression is reported in the standard ASTM D5045. One-factor analysis of variance (ANOVA) was performed on both the K_IC_ values and the impact energy before and after thermal mending to determine the correlations between the different variables. The null hypothesis was rejected if the F-value exceeded the critical F-value or if the *p*-value was <0.05 [33]. Additionally, Tukey’s test was performed to identify which groups of results were statistically different from one another [34].

#### 2.3.4. Evaluation of the Healing Efficiency

In the literature, the efficacy of the healing process is commonly described in terms of healing efficiency (HE), and is typically expressed as a relative percentage of the mechanical properties, such as strength, stiffness, and toughness [35]. In this study, thermal mending was performed on the prepared compatibilized blends. The specimens utilized in fracture toughness tests, both under quasi-static and impact conditions, were repaired thanks to the use of a lab-made device comprehensively described in previous works of our group [30,31], by putting them in an iron vice with an applied pressure of 0.5 MPa, and then heating them in an oven for 60 min at different temperatures (120, 140, 160, and 180 °C). The specimens subjected to thermal mending were tested again in both quasi-static and impact modes and the fracture toughness of the healed specimens ($K_{IC\_Healed}$) was obtained. Thus, the healing efficiency (${HE}_{KIC}$) was evaluated by using the expression reported in Equation (3),

(3) $${HE}_{KIC}=\frac{K_{IC\_Healed}}{K_{IC\_Virgin}}\;\cdot 100$$

where $K_{IC\_Virgin}$ and $K_{IC\_Healed}$ are the critical stress intensity factors of the virgin and healed specimens, respectively. Thus, it was possible to determine the influence of compatibilizer amount and healing temperature on the self-repair capability of the prepared blends.

## 3. Results and Discussion

**Figure 1:**
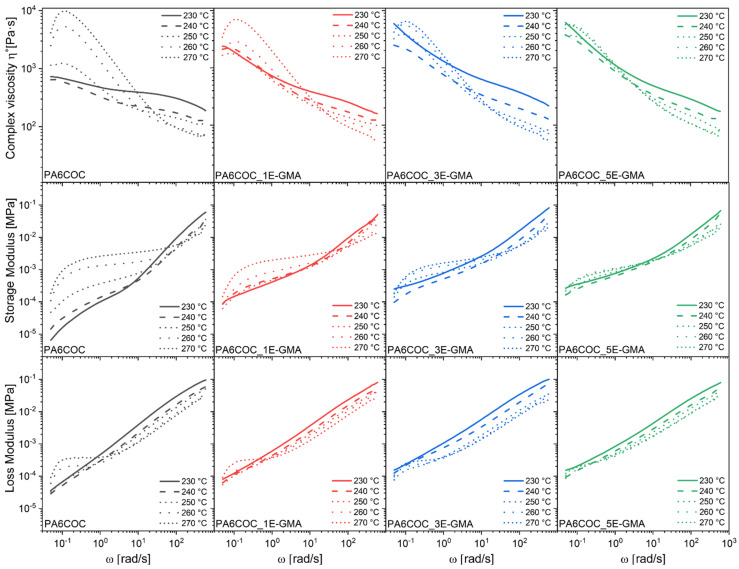
Dynamic rheological behavior (complex viscosity, storage modulus, and loss modulus) as a function of temperature of the produced PA6/COC/E-GMA blends.

A comprehensive analysis was performed to investigate the rheological properties of the produced blends and evaluate their processability and miscibility upon compatibilization. The results of the dynamic rheological measurements (Figure 1) highlight that the incorporation of E-GMA in the PA6/COC blend increases the complex viscosity over the entire frequency range, particularly in the low-frequency interval. This trend is particularly evident in the curves obtained at lower temperatures, where a more pronounced shear-thinning behavior is observed with increasing E-GMA content. This increment is the result of the improved interfacial interaction between PA6 and COC owing to the presence of the compatibilizer. The epoxide rings of E-GMA chemically react with the amine end groups and/or amidic bonds of PA6. The resulting PA6-EGMA copolymers, which can interact with both PA6 and COC, lead to a considerable improvement in adhesion and better dispersion of the COC domains [36].

Regarding the dependence of the complex viscosity on temperature, the viscosity decreases when the temperature rises from 230 °C to 240 °C, and then it increases again at 270 °C, particularly in the uncompatibilized blend. A similar trend has been reported by Radlmaier et al. [37], who observed a significant increase in the complex viscosity of PA6 at high temperatures during exposure to specific temperature profiles in air. They attributed this to the thermo-oxidative degradation mechanism, which involves the formation of reactive end groups via chain scission. At high temperatures, different chemical reactions can occur, i.e., a thermal-only breaking of the chain linking the amide (CONH_2_) and alkyl group (CH_2_)_5_; if water is present in the system, the chain linking the carboxyl (COOH) and amine (NH_2_) groups in the recurring amide group (CO-NH) is hydrolyzed [38,39]. During rheological tests, PA6 was held at high temperatures for a prolonged period, and both oxygen and moisture could diffuse within the specimens, initiating post-condensation and crosslinking reactions, and increasing the viscosity. This viscosity increase is more pronounced in the uncompatibilized blend, where a markedly non-Newtonian behavior, involving the amplification of the shear-thinning, can be observed with increasing temperature. However, this effect is mitigated by increasing the compatibilizer content. This behavior may be explained by the fact that the shorter molecular distances in the uncompatibilized blend allow easier diffusion of reactive species within the material [37].

Figure 1 also shows the trends in the dynamic moduli (G′, G″) at 230–270 °C. The addition of E-GMA to the PA6/COC system increases G′ over the entire frequency range and is more evident at low frequencies. Shin et al. [40] similarly reported that adding glycidyl methacrylate (GMA) in PA6 matrices significantly elevates the storage modulus compared to virgin PA6, with a three-order magnitude increase in G′ at low frequencies. This increase was attributed to the compatibilization reaction between PA6 and GMA, altering molecular structure through grafting reactions. Similar to what is observed for complex viscosity, G′ and G″ also rise at higher temperatures, especially in uncompatibilized blends, likely due to branching/chain scission from thermo-oxidative degradation.

**Figure 2:**
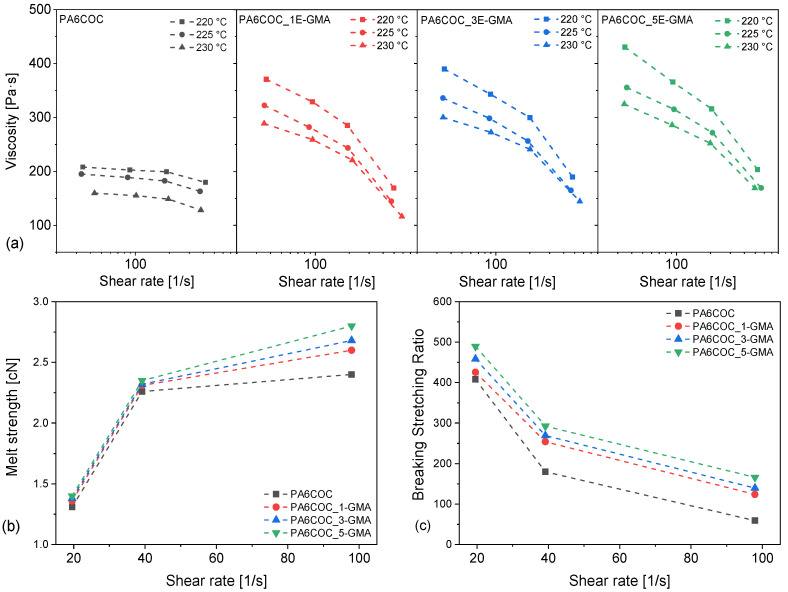
Capillary rheological measurements of PA6/COC/E-GMA blends. (**a**) Viscosity as a function of the shear rate at different temperatures, (**b**) melt strength at 230 °C, and (**c**) breaking stretching ratio (BSR) at 230 °C as a function of the shear rate.

Dynamic rheological tests were complemented by capillary rheological measurements to better understand the processability of the investigated blends under extrusion. The results of the capillary rheometer tests are shown in Figure 2a–c, which show the viscosity of the prepared blends as a function of the shear rate, at three different temperatures (220, 225, and 230 °C), and the melt strength and BSR values at 230 °C, that is, the temperature adopted in the last step of the extrusion process. From Figure 2a, it is evident that the viscosity measured using the capillary rheometer is significantly lower than that obtained with the rotational viscometer. This discrepancy can be explained by the presence of convergent flow at the inlet of the capillary, which deforms the geometry of the dispersed phases, thereby decreasing the viscosity of the melt [41]. The flow behavior of the prepared blends is a combination of reversible elastic deformation due to molecular entanglement and irreversible viscous flow due to polymer chain slippage.

By increasing the E-GMA concentration, the viscosity values at different shear rates tend to increase, and the non-Newtonian behavior of the blends becomes more pronounced [42,43]. This is in good agreement with the trends of complex viscosity and storage modulus measured with dynamic rheological measurements (Figure 1), highlighting the compatibilization effect provided by E-GMA. This effect was also observed in previous studies. For example, Scaffaro et al. [42] employed an ethylene–acrylic acid (EAA) copolymer to compatibilize low-density polyethylene (LDPE)/PA6 blends and demonstrated grafting of EAA with PA6, leading to the production of EAA-g-PA6 graft copolymers. The compatibilization was mainly due to the migration of the EAA-g-PA6 copolymers to the PA6-LDPE interface, which enhanced the adhesion between the two phases and, thus, the viscosity of the melt [43].

Figure 2b shows the rheological results in terms of melt strength, which describes the extensional load at the melt strip fracture. It is widely accepted that melt strength is a crucial parameter for polymers processed through spinning, blow molding, and foaming [44] because it measures the ability of the polymer melt to withstand elongational deformation. Typically, an increase in the melt strength indicates an improvement in the load-bearing capacity of the polymer melt, which, in turn, simplifies the extrusion process. The melt strength of a material depends on the molecular chain entanglements and resistance to untangling under strain, which is mainly enhanced by an increase in molecular weight, molecular weight distribution, and chain branching. In particular, branched polymers require greater strain to untangle the molecules and induce flow, whereas linear polymers can be more easily untangled when an elongational strain is applied. Figure 2b clearly shows that the melt strength of the compatibilized blends is significantly higher than that of the uncompatibilized blends. This is, again, due to the compatibilization induced by E-GMA [32] and the production of PA6-EGMA copolymers, which also enhances the processability of these blends by extrusion.

Moreover, the production of thin films requires a high BSR because the melt experiences a significant reduction in thickness during processing. Hence, only melts with greater deformability can be easily blown or extruded into films and are suitable for production processes such as film stacking, which is typical of thermoplastic composites reinforced with continuous fibers. As shown in Figure 2c, the compatibilized blends are characterized by higher BSR compared to the uncompatibilized blend, which confirms their enhanced processability in processing techniques that involve non-isothermal elongational flow, such as film-blowing operations.

**Figure 3:**
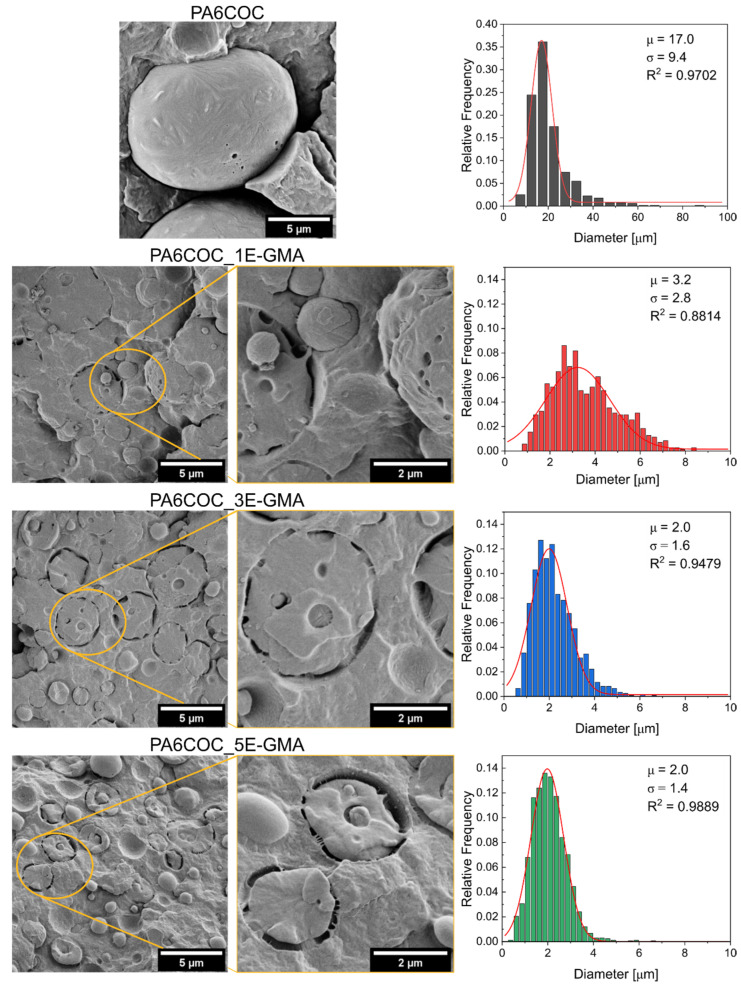
FESEM micrographs of the prepared PA6/COC/E-GMA blends and the relative diameter distribution of the COC domains together with the lognormal fitting results.

The phase morphology of the prepared blends, and the effect of E-GMA compatibilization, were investigated through FESEM, and Figure 3 reports the micrographs of the cryofracture surface of the prepared samples. The uncompatibilized blend is characterized by evident phase separation, as expected from non-miscible polymers [45,46], with COC domains showing little interfacial interaction with PA6. On the other hand, the introduction of E-GMA leads to a significant decrease in the COC domain size and a strong improvement in interfacial adhesion. As previously explained, E-GMA can chemically react with PA6, which enhances the compatibility of blends between PA6 and olefins, such as polyethylenes and polypropylenes [47,48]. Although E-GMA can chemically react with PA6, it can only entangle and form weak intermolecular bonds with COC [49]. These results are consistent with those obtained from dynamic rheological and capillary measurements (see Figure 1 and Figure 2 a–c).

Figure 3 shows the COC diameter distribution of the prepared blends. For the uncompatibilized blend, large COC domains, with diameters of approximately 20 µm, were observed. However, when E-GMA is added, the COC domains become much smaller, with a mean size of approximately 2 µm and a much narrower size distribution. An increase in the E-GMA content to 3 wt% and 5 wt% does not decrease the average domain size but it does further narrow the distribution. This significant decrease in the COC domain diameter and improvement in the COC/PA6 interaction demonstrates the effectiveness of E-GMA as a compatibilizer.

**Figure 4:**
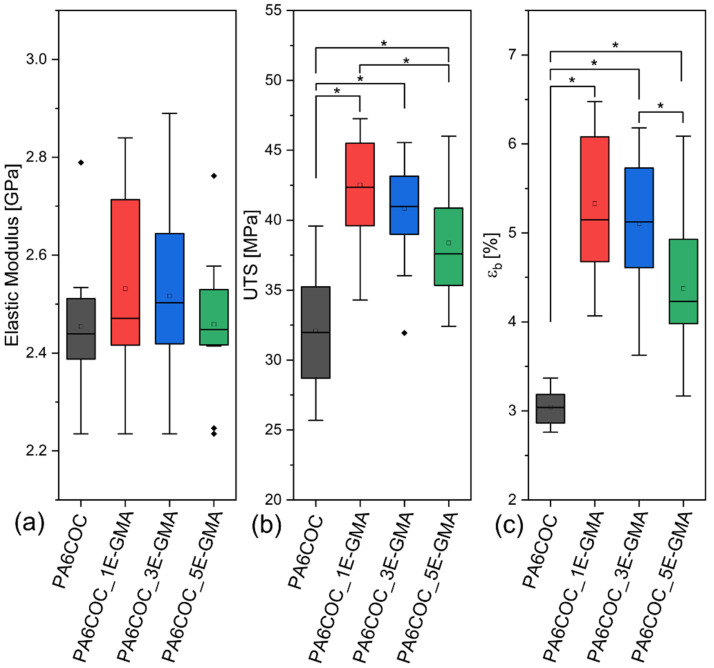
Results of tensile tests performed on PA6COC compatibilized blends. (**a**) Elastic modulus, (**b**) UTS, and (**c**) strain at break. * denotes statistically significant differences. Dots represent outliers.

The effective compatibilization of E-GMA on the prepared PA6/COC blends was investigated by measuring their mechanical properties through tensile tests. As shown in Figure 4, the addition of E-GMA does not significantly affect the elastic modulus of the prepared blends, as evidenced by the results of the one-way ANOVA and Tukey’s tests. On the other hand, the addition of 1 wt% E-GMA significantly increases both the ultimate tensile strength and strain at break, indicating effective compatibilization. Further increase in the E-GMA content does not lead to further improvements in the mechanical properties. Indeed, samples with an E-GMA content of 5 wt% perform slightly but significantly worse than those containing 1 wt% or 3 wt% E-GMA in both UTS and ε_b_, although all the compatibilized blends perform significantly better than the PA6COC uncompatibilized composition. The enhancement of the mechanical properties is correlated to the improved interfacial adhesion and decrease in the COC domain size, as shown in the FESEM micrographs (see Figure 3). The selected compatibilizer is capable of reacting with PA6, enhancing blend miscibility by lowering the surface tension of PA6 [50].

**Figure 5:**
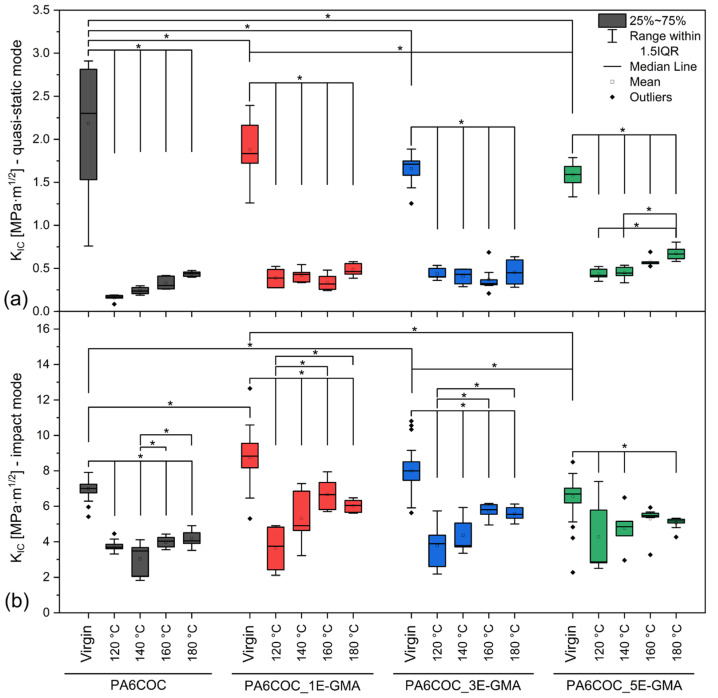
Fracture toughness of the non-compatibilized and compatibilized blends before and after the healing process. (**a**) Quasi-static mode and (**b**) impact mode. “Virgin” refers to the unbroken samples. * denotes statistically significant differences.

Figure 5 shows the trends of K_IC_ for the prepared blends, tested in quasi-static and impact modes, for the virgin (unbroken) and healed samples. The results of the two-way ANOVA and Tukey’s test are also reported, with statistically significant differences highlighted with asterisks. Looking at the results of the quasi-static tests for the virgin samples (Figure 5a), a slight decrease in K_IC_ can be observed with an increase in the E-GMA content. This effect has already been reported in the literature [51,52]. For example, Chiou et al. [51] prepared PA6/poly(phenylene ether) (PPE) blends (50:50 *wt*:*wt*) compatibilized with 5 phr of poly(styrene-co-maleic anhydride) (SMA). Although they observed an increase in elastic modulus, tensile strength, and strain at break in the compatibilized blend, they witnessed a slight decrease in K_IC_, measured on SENB specimens in the quasi-static mode. Since the aim of this study was to investigate the fracture behavior of PA6/PPE/SMA samples as a function of the content of an elastomeric phase, they did not deepen the investigation of the differences between the uncompatibilized and compatibilized base blends and did not advance any hypotheses on the reason for the decrease in fracture toughness. In this case, as for the present work, the reason may lie in the fact that the finer dispersion and increased interfacial adhesion in the compatibilized blends, although increasing the tensile properties, may also lead to a more complex fracture mechanism, potentially resulting in lower K_IC_ values, owing to less effective energy dissipation during crack propagation. Nevertheless, the effect of E-GMA on K_IC_ is minor.

For the healed samples, the K_IC_ increases with the E-GMA content and the healing temperature, and so does the healing efficiency (Table 2). This occurs because E-GMA improves the dispersion of COC and the interfacial interaction with PA6, but also because E-GMA can react and create covalent bonds with PA6, whereas the interactions with COC are likely mostly weak intermolecular bonds and physical entanglements, as evidenced in our previous work [32]. Hence, COC is still free to move and flow during healing, and as expected, its mobility increases with the healing temperature. The highest healing efficiency in quasi-static mode is measured for the sample with the highest amount of compatibilizer (PA6COC_5E-GMA) healed at the maximum healing temperature (180 °C). Still, the maximum healing efficiency is quite low, being equal to only 41 ± 4%.

The situation changes considerably under the impact conditions (Figure 5b). For the virgin samples, the results indicate a slight but significant increase in K_IC_ with the addition of up to 3 wt% E-GMA, followed by a slight decrease at 5 wt%. In the case of healed samples, the K_IC_ values are predominantly influenced by the healing temperature, but the increase in E-GMA also gives a beneficial contribution. The HE (Table 2) increases with the compatibilizer content and healing temperature, but up to 160 °C, after which a slight decrease is observed. Notably, the value of the impact K_IC_ of PA6COC_5E-GMA healed at 160 °C is not significantly different from that of virgin, unbroken PA6COC_5E-GMA. The highest healing efficiency in impact mode is measured for this sample, i.e., PA6COC_5E-GMA healed at 160 °C, and is equal to 82 ± 4%.

Overall, these results demonstrate that the E-GMA compatibilizer plays a significant role in enhancing the fracture toughness and self-healing capabilities of PA6/COC blends. The improved repair ability of the compatibilized blends is likely due to the enhanced interaction between the COC dispersed phase and the PA6 matrix, as evidenced by rheological and morphological analyses, combined with the retained flowability of COC. However, the healing efficiency values in the impact mode are always two or three times higher than those measured in the quasi-static mode (Table 2). This interesting effect can be attributed to the distinct fracture surface morphologies produced by the different testing speeds, as supported by the SEM micrographs shown hereafter.

**Figure 6:**
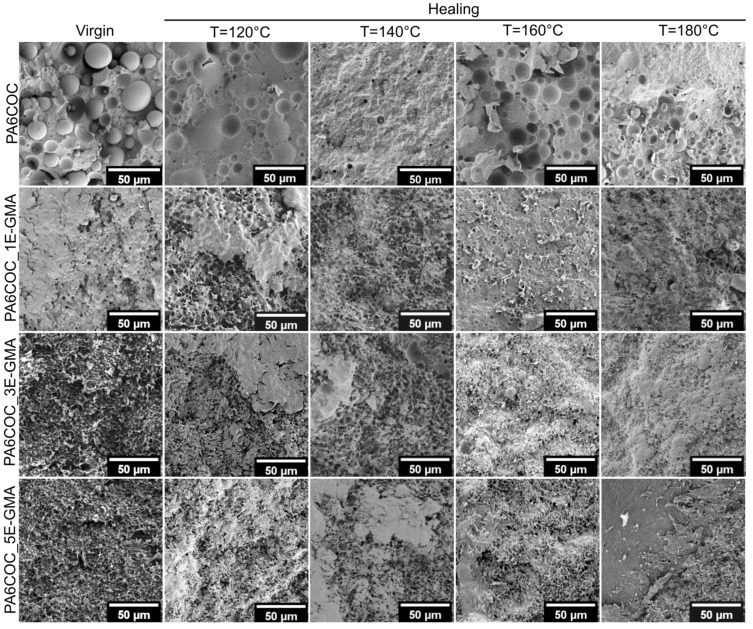
FESEM micrographs of the fracture surface of the PA6/COC compatibilized blends before and after the healing process, tested in quasi-static mode.

**Figure 7:**
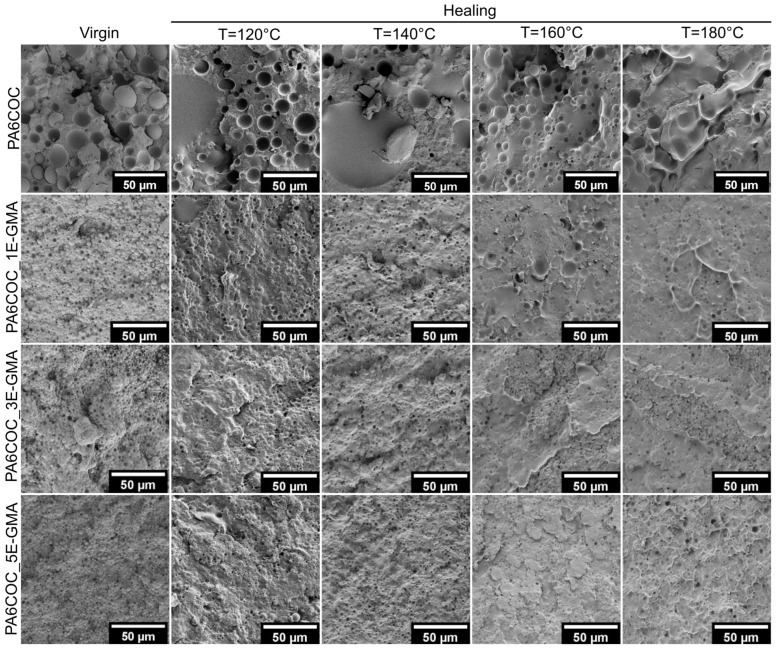
FESEM micrographs of the fracture surface of the PA6/COC compatibilized blends before and after the healing process, tested in impact mode.

The differences in the healing efficiency measured in the quasi-static and impact conditions can be explained by examining the fracture surfaces of the virgin and healed samples, as shown in Figure 6 (quasi-static mode) and Figure 7 (impact mode). For the samples tested in quasi-static mode, Figure 6 clearly shows that upon the introduction of E-GMA, virgin samples exhibit substantial plasticization in the PA6 matrix, with the generation of a peculiar morphology (see first column in Figure 6). As the healing process occurs, the COC domains soften and migrate toward the cracked area. It becomes apparent that the COC tends to remain isolated in particular regions, rather than filling the crack completely. Consequently, the substantial plasticization of the PA6 matrix, which results in an uneven crack surface, hinders the COC from effectively sealing the cracks. This ultimately leads to low HE values. Nevertheless, E-GMA also reduces the surface tension of PA6, resulting in smaller COC domains with a superior interfacial interaction with the surrounding PA6 matrix. Under these conditions, the COC can flow within the crack zone more efficiently. By increasing the healing temperature, it is possible to notice that the COC domains tend to spread and fill the damaged surface more effectively, which explains why the healing efficiency in quasi-static mode tends to increase with healing temperature, although it remains modest.

The crack morphology considerably changes in impact conditions (Figure 7). Because the fracture surface is smoother and more regular than that observed in the quasi-static mode, the COC domains can fill the crack plane. Interestingly, by performing thermal mending at 160 °C and 180 °C, it appears that the healed surfaces are covered by a thin and uniform COC film. Furthermore, the improved interfacial interaction between COC domains and PA6 contributes to a further increase in the HE values.

This research demonstrates that the shape and structure of the crack surface significantly impact the effectiveness of the healing agent. Additionally, it shows that the chemical and physical interactions occurring at the crack interface play crucial roles in determining the overall success of the repair process. In fact, we claim that fractography analysis is crucial for advancing the understanding of the self-healing performance of materials. Although healing efficiency metrics, such as the percentage of mechanical strength recovered, offer a numerical measure of repair capability, they cannot explain the intricate details of the healing process itself.

However, the establishment of a standardized approach for fractography analysis in the context of self-healing materials remains pending, which represents a significant gap in the field of self-healing materials. The literature has reported various techniques for evaluating healing efficacy, ranging from mechanical testing to microscopic evaluation. However, direct comparisons between these methods remain challenging due to the absence of a standardized framework. Establishing such a standard would not only enhance the reliability and comparability of research findings but also accelerate the development and optimization of self-healing materials with superior performance characteristics.

## 4. Conclusions

This work demonstrated the successful formulation of PA6/COC/E-GMA blends, which possess the potential to function as self-healing matrices for thermoplastic structural composites. A comprehensive examination of the rheological, thermal, and mechanical properties of the blends demonstrated the positive influence of E-GMA on enhancing the repair capabilities of the system.

The rheological behavior of the blends, particularly the trends of viscosity, melt strength, and breaking stretching ratio as a function of shear rate, revealed the compatibilizing effect of E-GMA. Although E-GMA increased the melt viscosity, it significantly improved MS and BSR, revealing a better film and fiber-forming potential. The compatibilization effect of E-GMA was also proven via microstructural and mechanical analysis, which revealed that E-GMA effectively decreased the COC domain size, improved the interfacial interaction with the surrounding PA6 matrix, and improved the tensile strength and strain at break.

The quasi-static and impact fracture toughness evaluations revealed that the addition of E-GMA did not increase the K_IC_ values but significantly enhanced the healing efficiency of the system. The results of this extensive experimental campaign indicated that the optimal healing temperature for the blends was 160 °C. In the quasi-static mode, the healing efficiency of the uncompatibilized blend healed at 160 °C was 12%, whereas that of PA6COC_5-EGMA was 38%. When considering the results in the impact mode, uncompatibilized PA6COC reported an HE value of 57%, while that of PA6COC_5-EGMA was 81%. The reason for these large differences in the quasi-static and impact modes was determined by examining the fracture surfaces. In the quasi-static mode, the presence of E-GMA caused severe plasticization, which hindered the optimal flow of healing agents within the crack zone. In contrast, under impact conditions, the fracture surface was more planar, allowing the healing agent to flow and fill the crack plane properly, leading to higher repair capabilities.

Overall, this work demonstrates the potential of E-GMA for tuning the thermomechanical properties and healing capability of PA6/COC blends. From this analysis, PA6COC_5E-GMA emerged as the optimal system with the best balance between self-healing efficiency and processability. This formulation could thus be utilized as a matrix in thermoplastic structural composites with good repair capabilities, which will be the subject of future investigations.

## Figures and Tables

**Table 1:** List of prepared samples with nominal relative amounts of constituents.

Sample	PA6 Content [wt%]	COC Content [wt%]	E-GMA Content [wt%]
PA6COC	70.0	30.0	0.0
PA6COC_1E-GMA	69.3	29.7	1.0
PA6COC_3E-GMA	67.9	29.1	3.0
PA6COC_5E-GMA	66.5	28.5	5.0

**Table 2:** The healing efficiency [%] of the prepared blends calculated from the K_Ic_ values in the quasi-static and impact modes.

Quasi-Static Mode				
Sample/T healing (°C)	120	140	160	180
PA6COC	8 ± 3	10 ± 3	12 ± 3	17 ± 2
PA6COC_1E-GMA	17 ± 5	19 ± 3	20 ± 4	30 ± 7
PA6COC_3E-GMA	25 ± 4	24 ± 5	23 ± 7	29 ± 8
PA6COC_5E-GMA	27 ± 4	28 ± 4	38 ± 3	41 ± 4
**Impact mode**				
Sample/T healing (°C)	120	140	160	180
PA6COC	46 ± 7	45 ± 9	57 ± 5	58 ± 6
PA6COC_1E-GMA	48 ± 12	54 ± 9	75 ± 3	69 ± 3
PA6COC_3E-GMA	54 ± 17	50 ± 14	73 ± 5	70 ± 1
PA6COC_5E-GMA	67 ± 22	74 ± 16	82 ± 4	74 ± 5

## Data Availability

The original contributions presented in this study are included in the article. Further inquiries can be directed to the corresponding authors.
